# Dr. Mazet

**Published:** 1822-02

**Authors:** 


DR. MAZET.

This young physician, whose death we announced in another Number, was one of those intrepid and enlightened practitioners, who, braving the perils of a destructive epidemic, flew to the assistance of the wretched population of Barcelona, with four others of his countrymen, when danger was at its height; and there fell a lamented victim to his thirst for knowledge, and his undaunted zeal in the cause of suffering humanity. In a subsequent Number, we had the gratification of announcing that the King of France had approved of a subscription, set on foot by the medical students in Paris, for erecting a monument to the memory of that distinguished and amiable practitioner; and we feel great pleasure in adding, that, on the suggestion of the Chamber of Deputies, a mark of royal favour, in the form of a pension, will, it is expected, be conferred on the mother and sisters of the deceased. Professor Desgenettes, being desirous to testify his sense of the loss which the friends of Dr. Mazet, and the profession in general, have sustained, has proposed the following, as a suitable inscription, to be engraved on his sepulchral monument:

ANDREÆ MAZET

D. M. P.

INTER CONTAGIUM

BARCINOMI DEBELLANDUM

DE CORÂ MORTE

OCCUMBENTI

FACULTAS MEDICA PARISIENSIS

M.DCCCXXI.

